# Beef Tea

**Published:** 1903-01-03

**Authors:** 


### Editor's Letter-Box.

[Our Correspondents are reminded that prolixity is a great bar to publication, and that brevity of style and conciseness of statement greatly facilitate early insertion.]

BEEF TEA.

S. H. writes:—The matron of the Cranworth Workhouse Infirmary, who writes to you in praise of Bovril, would do well to study "Food and the Principles of Dietetics," by R. Hutchison. There on pp. 94 and 95 she will see what Bovril is made of and what its value is as a food. She will learn that a "full teaspoonful only contains as much proteid as one-fifth ounce of meat; it would require about 14 ounces of such a preparation to supply an invalid with the amount of proteid he requires daily, but such a quantity he would find it impossible to consume owing to the enormous quantity of salts and extractives which it would contain." And he further tells us that: *As a matter of fact the white of one egg will contain as much nutritive matter as three teaspoonfuls of any of the preparations such as Bovril.* On page 101 she will learn how to make really useful beef tea—a beef tea containing real nutriment. I write in the interest of the matron's patients.

[It ought to be added that, even in regard to this "really useful beef-tea," Dr. Hutchison says that "it can never be regarded as an important aid in the feeding of the sick. If one swallows a pint of it in a day, he has only consumed about one-ninth of the total amount of proteid required by a sick person."—EDITOR OF THE HOSPITAL.]

